# Molecular Mechanism of Allosteric Communication in Hsp70 Revealed by Molecular Dynamics Simulations

**DOI:** 10.1371/journal.pcbi.1002844

**Published:** 2012-12-27

**Authors:** Federica Chiappori, Ivan Merelli, Giorgio Colombo, Luciano Milanesi, Giulia Morra

**Affiliations:** 1 Istituto di Tecnologie Biomediche – Consiglio Nazionale delle Ricerche (ITB-CNR), Segrate (Mi), Italy; 2 Istituto di Chimica del Riconoscimento Molecolare - Consiglio Nazionale delle Ricerche (ICRM-CNR), Milano, Italy; University of Wisconsin, Madison, United States of America

## Abstract

Investigating ligand-regulated allosteric coupling between protein domains is fundamental to understand cell-life regulation. The Hsp70 family of chaperones represents an example of proteins in which ATP binding and hydrolysis at the Nucleotide Binding Domain (NBD) modulate substrate recognition at the Substrate Binding Domain (SBD). Herein, a comparative analysis of an allosteric (Hsp70-DnaK) and a non-allosteric structural homolog (Hsp110-Sse1) of the Hsp70 family is carried out through molecular dynamics simulations, starting from different conformations and ligand-states. Analysis of ligand-dependent modulation of internal fluctuations and local deformation patterns highlights the structural and dynamical changes occurring at residue level upon ATP-ADP exchange, which are connected to the conformational transition between closed and open structures. By identifying the dynamically responsive protein regions and specific cross-domain hydrogen-bonding patterns that differentiate Hsp70 from Hsp110 as a function of the nucleotide, we propose a molecular mechanism for the allosteric signal propagation of the ATP-encoded conformational signal.

## Author Summary

Allostery, or the capability of proteins to respond to ligand binding events with a variation in structure or dynamics at a distant site, is a common feature for biomolecular function and regulation in a large number of proteins. Intra-protein connections and inter-residue coordinations underlie allosteric mechanisms and react to binding primarily through a finely tuned modulation of motions and structures at the microscopic scale. Hence, all-atom molecular dynamics simulations are suitable to investigate the molecular basis of allostery. Moreover, understanding intra-protein communication pathways at atomistic resolutions offers unique opportunities in rational drug design. Proteins of the Hsp70 family are allosteric molecular chaperones involved in maintaining cellular protein homeostasis. These proteins are involved in several types of cancer, neurodegenerative diseases, aging and infections and are therefore pharmaceutically relevant targets. In this work we have analyzed, by multiple molecular dynamics simulations, the long-range dynamical and conformational effects of ligands bound to Hsp70, and found relevant differences in comparison to the known non-allosteric structural homolog Hsp110. The resulting model of the mechanism of allosteric propagation offers the opportunity of identifying on-pathway allosteric druggable sites, which we propose could guide rational drug-design efforts targeting Hsp70.

## Introduction

Heat shock proteins (HSPs) are essential macromolecules involved in housekeeping cellular activities, whose expression levels can be modulated in response to environmental conditions. The Hsp70 family of proteins plays essential roles in maintaining cellular protein homeostasis. Under normal conditions, Hsp70 can fold nascent polypeptides as they emerge from ribosomes or refold misfolded proteins, regulate the stability and activity of specific proteins and solubilize aggregates [1], [2]. Hsp70 is also involved in protein degradation, ubiquitination, assembly and disassembly of oligomeric complexes and translocation of proteins across membranes [2], [3], [4]. Under stress conditions, increased expression of Hsp70 helps to preserve and recover the correct functional structure of client proteins by binding to denatured conformations [1].

Given its involvement in many cellular control and regulation processes, recent studies have shown a key role of Hsp70 in several diseases: some of these, for instance several cancer types (breast, endometrial, oral, colorectal, prostate cancers, and certain leukemias) are associated with overactivity/overexpression of the chaperone [5]. Defects in Hsp70's activity and consequent abnormal protein misfolding and accumulation are involved in neurodegenerative diseases, such as Alzheimer, Parkinson, and Huntington [5], and in aging processes [6], [7]. This evidence points to Hsp70 as an interesting drug target [5], [7], [8].

**Figure 1:**
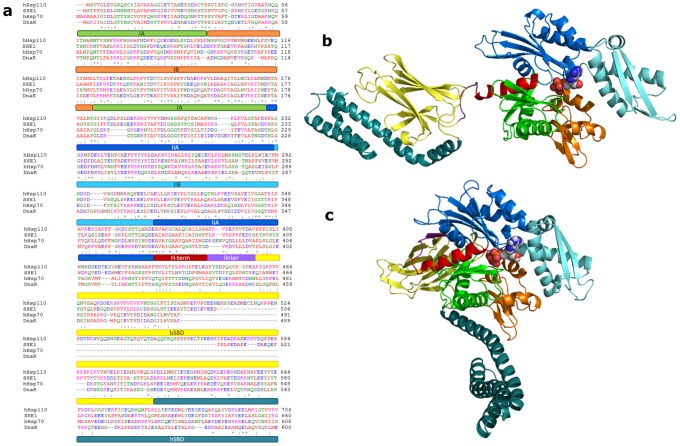
Protein sequences and structures. a) Sequence alignment of human Hsp110, yeast Sse1, human Hsp70 and bacterial DnaK. b) Crystal structure of ADP bound *closed* DnaK (2KHO.pdb); c) Homology model of ATP bound *open* DnaK based on Sse1 (3C7N.pdb). Subdomains are colored according to bars in a).

From the structural viewpoint, members of the Hsp70 family are composed of two domains connected by a highly conserved 14 residue-linker: a ∼44 kDa N-terminal nucleotide binding domain (NBD), with ATPase activity, and a ∼25 kDa substrate binding domain (SBD), which binds peptides [2], [4] (Figure 1). The NBD consists of lobe I and lobe II, which in turn can be divided into subdomains: IA (residues 1–37 and 120–171) and IB (residues 38–119), IIA (residues 172–227 and 311–368) and IIB (residues 228–310). Domains IB and IIB are connected by flexible hinges to IA and IIA respectively and regulate the access to the nucleotide binding site. The NBD terminal helix (residues 369–383) is localized between the two lobes and connects the NBD to the inter-domain linker. The SBD also contains two subdomains, a β-sandwich base (βSBD) and a domain made of 5 α-helixes (A to E) (αSBD) forming a lid over the polypeptide binding site [1], [9]. The βSBD loops protrude upwards forming a deep hydrophobic cavity closed up by helix B, where peptides can bind in a linear conformation [2].

In Hsp70 the relative three-dimensional arrangement of the two domains is regulated by the presence of a specific nucleotide and their activities are coupled through allosteric mechanisms: the specific nucleotide bound to the NBD regulates the SBD conformation required for peptide binding [10], [11]. The crystal structure of bacterial Hsp70, DnaK, in the ADP-bound *closed* conformation [9] displays the two domains completely separated by the linker in a flexible and extended solvent exposed conformation. In contrast, the X-ray structure of yeast Hsp110, a structural homolog of Hsp70, shows an ATP-bound *open* conformation [12]. In this conformation, the αSBD is widely open with respect to the βSBD [2], and the linker folds in a β-strand localized in a hydrophobic binding pocket between the IA and IIA subdomains of the NBD, thus docking the SBD to the NBD [1], [7]. In the ATP-bound state, association and dissociation rates for substrates are high, while substrates affinity is low. After ATP hydrolysis to ADP the affinity for substrates is high, while substrate exchange rate is low [2]. Experimental evidence has established the allosteric coupling between NBD and SBD and the essential role of the linker in this mechanism [10], [13]. The linker transduces allosteric signals in both directions: polypeptide binding in the SBD can also transmit changes to the NBD, increasing the ATP hydrolysis rate [1]. Allosteric coupling between the two domains is absent in Hsp110 [2], in spite of the high structural similarity of the two proteins. The molecular determinants for the presence or absence of allosteric coupling in these families of proteins are still poorly understood and they represent a significant challenge and an opportunity to structure-based drug design [14].

In this study, we aim at elucidating the atomic origins of the allosteric communication in Hsp70 protein family in comparison with non-allosteric Hsp110 by means of molecular dynamics (MD). By simulating several protein-nucleotide complexes in a fully solvated environment and applying a set of structural and dynamical analyses specifically developed for the study of allosteric systems [15], [16], [17], [18], we aim to gain insights into the mechanisms of nucleotide-induced signal propagation in Hsp70 and identify functional hotspots involved in the response to ATP and ADP in different conformational states of the protein. To this end, we simulated multiple MD trajectories of Hsp70 and Hsp110 proteins in complex with ATP, ADP and in the apo form (total simulation time: 1.9 microseconds). The all-atom detail is maintained throughout the analysis, with the aim of relating the observed large-scale motions and conformational changes to their atomistic physico-chemical origin. The comparison between the allosteric and the non-allosteric species allows determining the interactions and specific clusters of residues that are responsible for the different long-range, nucleotide-driven structural effects at the SBD domain. Finally, coarse-grained elastic network models (ENM) are used to investigate the conformational transition mechanisms, and the results are critically discussed with respect to the ones from all-atom simulations.

## Results

A comparative analysis of both Hsp70 and its non-allosteric homolog Hsp110 is carried out in order to identify the determinants of the allosteric mechanisms at the atomic level. The functional cycle of the protein entails a transition from the *closed* state in the presence of ADP (Figure 1B), represented by the crystal structure of ADP-bound DnaK (a representative member of the Hsp70 family, whose functional dynamics is allosterically regulated) [9], to the *open* state in the presence of ATP, represented by the ATP-bound conformation of yeast Sse1 (an Hsp110 family member, whose functional dynamics is not allosterically regulated) [12]. We have analyzed the dynamical properties both of Sse1 and of a homology model of the *open* structure of ATP-bound DnaK (Figure 1C). In the absence of a high resolution experimental structure, open Hsp70 can be modeled based on the available Hsp110 conformation, as proposed by Smock [19] and by Zuiderweg [20]. This assumption implies a high structural similarity between the open forms of DnaK and Sse1, which may not be guaranteed in solution. However, experimental analysis of the functional response of DnaK to targeted mutations designed on the basis of the homology model [19], combined with previous biophysical and biochemical data that identified common/conserved residues among different members of the Hsp70 family that are important for interdomain communication, supports the use of ATP-bound Sse1 as a template for the structure of ATP-bound DnaK [19], [21], [22], [23]. It is important to underline that results obtained from the MD simulations for this model provide only qualitative insights into the mechanism.

We have studied each protein in the apo, ATP- and ADP-bound states. The presence of ATP in the *closed* state structure is expected to switch on a set of conformational events that can eventually trigger the transition towards the *open* state. In a complementary way, ADP in the *open* state is expected to favor the transition to the *closed* conformation. Considering the complexity of the involved mechanism and the limitations of MD sampling, the simulated trajectories are not sufficient to warrant the observation of complete conformational transitions among different states. Therefore, our study focuses mainly on the characterization of the structural and dynamical changes stimulated by a given ligand in each state. We aim at detecting specific protein regions that respond to the nucleotide with a conformational rearrangement or a modulation of dynamics. The latter aspect can be captured by the analysis of long-range coordinated residue pair fluctuations and local distortions in the protein's contact patterns. The underlying hypothesis is that the combination of coordinated microscopic motions on the ns time scale might result in a macroscopic structural transition on longer time scales [24].

The structural analysis of the simulated systems is carried out by means of a cluster decomposition of the joint MD trajectories. In order to relate the structural changes to the dynamical modulation induced by the different ligands, the matrices of distance fluctuations are calculated, which describe the internal Cα-Cα coordination and report on the possible rigid-like character of specific substructure motions. To identify flexible hinges and rigid substructures involved in the signal transduction between ligand and SBD, the time-dependent geometric strain and the hydrogen bond networks are analyzed and presented in the following sections.

Structural parameters calculated from the MD simulation of the open structure of Hsp70 were first analyzed and compared to the analogous parameters for the other systems. The Root Mean Square Deviation (RMSD) values for open Hsp70 were in the same range as the ones observed in the trajectories started from the crystal structures (Figure S1). Moreover, no major distortion of the secondary structure elements was observed (Figure S2). These data allowed us to rule out relevant structural and dynamical artifacts due to the modeling process.

### Structural basis for nucleotide-mediated conformational transitions

#### *Closed* Hsp70 conformation

**Figure 2:**
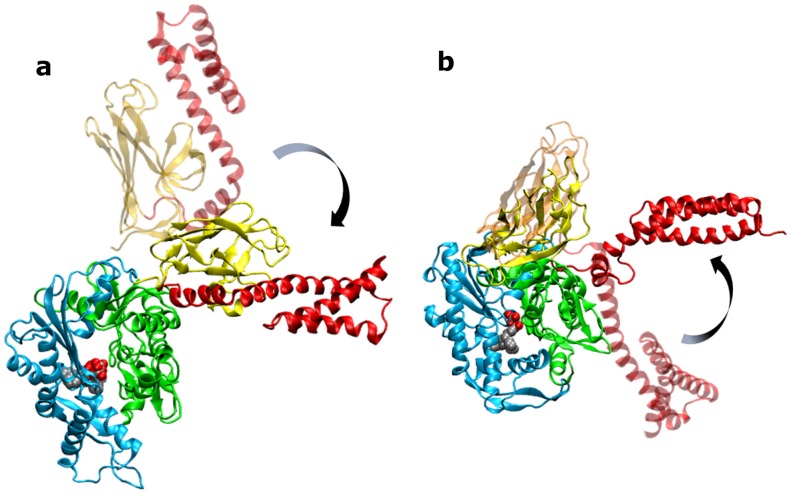
Open-close transition. a) MD simulation of ATP bound *closed* DnaK: final snapshot of a single MD run (100 ns, solid color) superimposed to the starting structure (transparent color), showing structural evolution of the αSBD and βSBD. b) MD simulation of ADP bound *open* DnaK: final snapshot of a single MD run (100 ns, solid color) superimposed to the starting structure (transparent color), showing structural evolution of the αSBD towards a *semi-closed* conformation. The conformational changes are indicated with an arrow.

**Figure 3:**
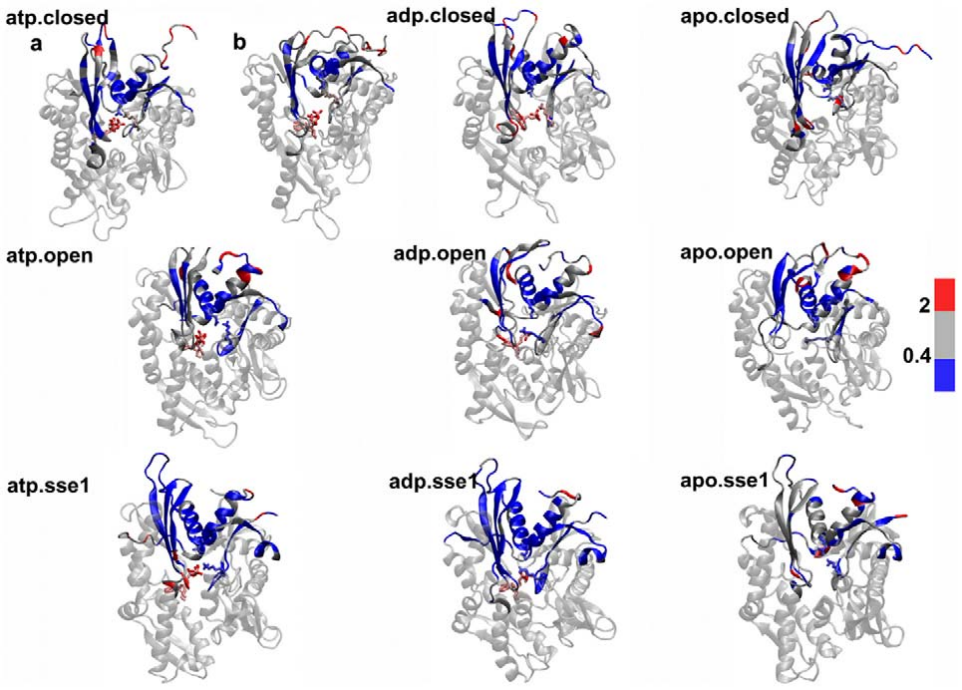
Nucleotide binding site. Most representative conformations of the ATP-binding site obtained by clustering on nucleotide binding site. The binding site residues, as well as loop 210 and the C-terminal helix and linker (residues 370–390) are color-coded according to the average local flexibility calculated over the MD trajectories: blue, local flexibility below 0.4 Å^2^; red, local flexibility above 2 Å^2^. For the ATP bound closed DnaK complex, two representative conformations are shown, namely the most populated one (a) and the alternative one showing loop 210 folding towards the linker (b), see main text for further details.

Starting from the initially *closed* conformation of DnaK, the presence of ATP instead of ADP determines a number of structural changes related to the onset of the conformational transition towards the *open* state (Figure 2a). Such changes entail the docking of βSBD onto the NBD, observed in all ATP-bound trajectories within 20–35 ns, and local unfolding at the helix turn that is expected to open, which occurs in two cases, after 20 ns and 95 ns, respectively. At the local level, the observed conformational change is accompanied by a perturbation of the binding site geometry (Figure 3). In the starting crystal structure, which is stable with ADP and still significantly populated with ATP in all trajectories, (cluster a, Figure 3), the catalytically relevant K67 and E168 are coordinated to the nucleotide, but loop 210 shows enhanced flexibility with ATP, in contrast to the ADP simulations. In the ATP-induced alternative state (cluster b, Figure 3) loop 210 folds towards the C-terminus of the NBD close to the linker, which is observed together with the onset of the opening motion at the SBD. At the same time, loop 195 (comprising residues 193–196) and loop 7–10, both in contact with the nucleotide, together with the β-sheets connecting loop 195 to loop 180 and 210, respond to ATP with increased structural rigidity with respect to the ADP bound and, even more, to the apo system. This results in a reduced mobility of the nearby hinge 222–225 and subdomain IIB (Figure 3) when ATP is bound.

The atomic coordination between each nucleotide and binding site residues induces a ligand-dependent relative arrangement of the DnaK four subdomains. Namely, in the presence of ATP, the most populated conformation (cluster b, Figure 3) shows a marked closure of the binding pocket, with subdomains IIB and IB approaching and forming a stable interaction between H223 and S84, which is absent in the ADP-bound and apo systems.

**Figure 4:**
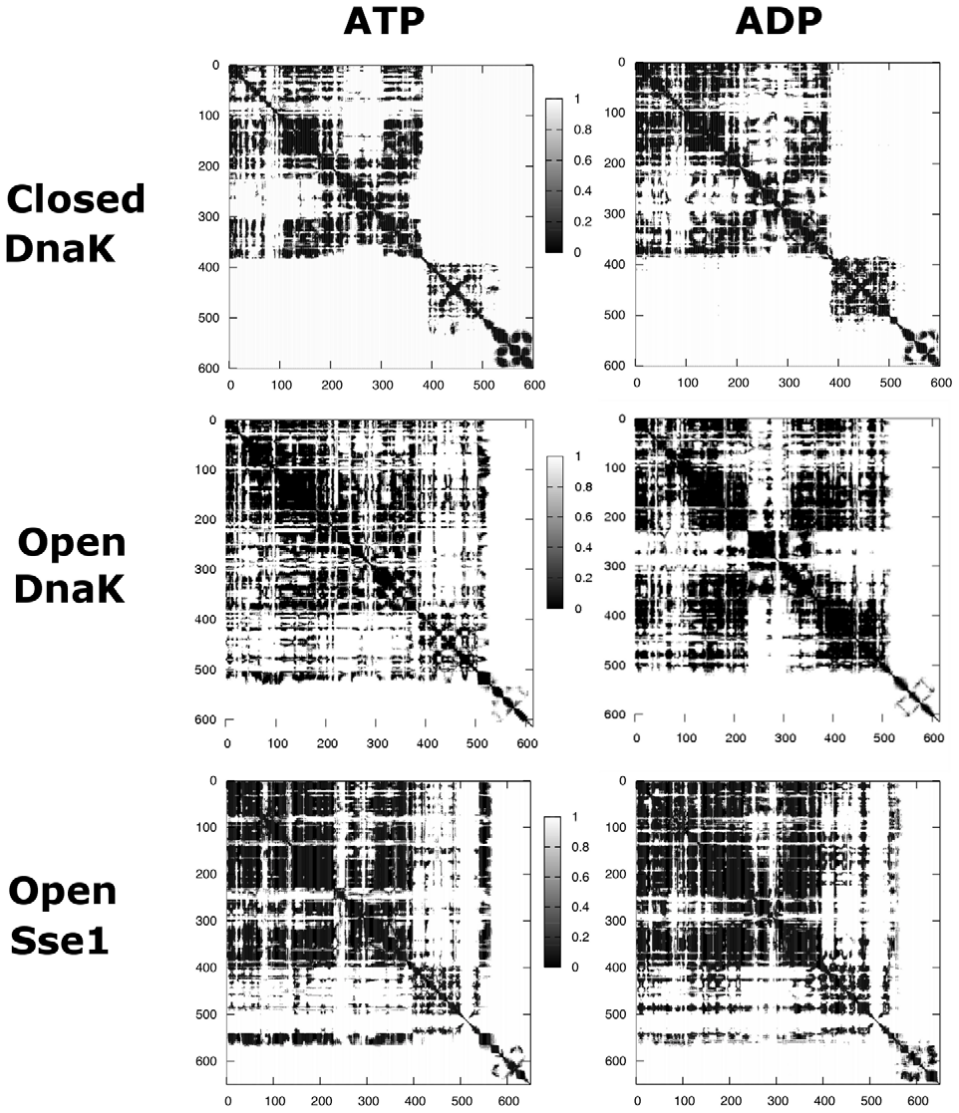
Map of distance fluctuations. Matrix of distance fluctuations calculated and averaged over the three MD trajectories for each ATP complex (left) and each ADP complex (right). Matrix entries are colored black if below 0.3 and white if above 0.3 Å^2^. For the distance fluctuation matrices referring to each individual MD run, see Supplemental Information (Figure S4).

The nucleotide-dependent conformational events at the binding site of the *closed* state reverberate also in the modulation of the rigid-body like motions of the four NBD subdomains. The average distance fluctuation matrix (Figure S3) of apo protein shows a significant block character and can be decomposed in four semi-rigid regions, corresponding to the four subdomains. In particular, a relative rigid motion of subdomains IIB and IIA and correspondingly pronounced flexibility at the connecting hinges are observed. In this context, subdomain IIB explores different orientations and the helical hinge around residue 305 (connecting IIA and IIB) undergoes local unwinding. In contrast, both ligands determine increase of inter-domain rigidity (Figure 4). With ATP, the enhanced rigid coordination between subdomains IIA and IIB is an effect of the stabilizing interaction of loop 195 (IIA) with the γ-phosphate group of the nucleotide, that in turn coordinates hinge 222–225 connecting subdomain IIA to IIB. Subdomain IB remains flexible, while an overall rigid coordination between IA and IIA is established. In the ADP complex (Figure 4) subdomain IA is rigidly coordinated to IB and they move coherently with respect to lobe II and to the lobes interface. On the other hand, the more open NBD conformation explored by the system is due to the higher conformational freedom of subdomain IIB, which moves around its two hinges (residues 225 and 308) with respect to domain IIA. Here the interface between subdomains IA and IIA at the linker-binding cleft is rather flexible and no persistent contacts between loop 210 and the linker are found.

Overall, we conclude that in the *closed* DnaK conformation, the ligand modulates two main structural and dynamical features of the NBD, depending on the binding site arrangement:

1. The closure of the clamp defined by lobe I and lobe II around the nucleotide binding site, which depends on the orientation and mobility of subdomain IIB relative to IB and is induced by the coordination of nucleotide and loop 195.
2. The conformation and rigidity of the binding cleft defined by subdomains IA and IIA and involved in binding the linker connecting NBD and SBD, which is modulated by loop 210.

Interestingly, the effects of different nucleotides diffuse to the SBD. From the dynamical point of view (Figure 4), the mobility pattern in the SBD is markedly ligand dependent. The inter-strand rigidity of the βSBD is reduced when ATP is bound compared to ADP, due to an increase in the flexibility of inner loops around 400 and 500. The βSBD of the apo system shows an intermediate rigidity between the ATP- and the ADP-bound protein (Figure S3). Notably, these dynamical effects are consistently observed in all MD runs, despite (expected) differences in the time evolution of specific transitions (Figure S4 A).

The most representative structures, obtained by cluster analysis based on the DnaK segment 395–600 for the ATP and ADP states, differ in the relative orientation of the αSBD and of the βSBD (Figure S5, Table S1). In the presence of ADP, the αSBD folds onto the βSBD, due to an enhanced bending of helix B with respect to helix A around residue 520, which increases the closure of the empty-peptide binding pocket. In contrast, when ATP is bound, the orientation of the αSBD diverges with respect to the βSBD, resulting in an opening of the pocket. The distance between helix B and subdomain IIA is reduced by about 6 Å in the cluster representative of trajectory 3.

#### *Open* Hsp70 conformation

The αSBD of the modeled *open* DnaK interacts extensively with NBD subdomains IA and IB, while βSBD is docked to NBD subdomain IA. Helix 80–90 in IB has a different orientation with respect to the *closed* state, resulting in a more open nucleotide cleft. In this case, the presence of ADP is expected to trigger the transition towards the *closed* state, leading to the experimentally observed conformation of ADP-bound DnaK. Indeed, the analysis of the MD trajectories of ADP-bound *open* DnaK shows a rearrangement of the βSBD and of the long SBD helix A–B (Figure 2), which in two out of three trajectories leads to an intermediate *semi-closed* conformation. The latter represents about the 22% of the conformations obtained from the whole trajectory clustering. Here, the detachment of the αSBD, its unfolding and the formation of a turn at residue 520, which induces the splitting of the long helix, is coupled to a re-orientation of the βSBD, which rotates towards the lower part of NBD-IB (Figure 2). The RMSD between this trajectory undergoing the *open-to-closed* transition (induced by ADP) and the aforementioned trajectory of *closed-to-open* DnaK transition (induced by ATP) decreases during simulation time (Figure S6). This indicates the convergence of two different structures to a similar intermediate conformation. In the remaining ADP-bound trajectory, structures show an initial detachment of the αSBD and its local unfolding. In contrast, in the ATP state the *open* structure is stable during the whole trajectory in all simulated cases: the βSBD remains docked to the NBD, while the αSBD keeps a constant orientation, with residue 530 contacting lobe I of NBD. Interestingly also in the apo state, the αSBD occasionally explores *semi-closed* conformations as well as *open* ones. Here, the βSBD occupies an intermediate position between the extreme cases of ATP and ADP.

Consistent with the previous case, global rearrangements are correlated to the structural dynamics of the binding site. In the ATP-state, the nucleotide stably interacts with loop 195 (Figure 3) in all MD trajectories. The overall shape of the binding pocket resembles that of cluster b (Figure 3) observed in the ATP-bound *closed*-DnaK simulation, with loop 210 stably bent towards the linker. The latter interaction is weakened by ADP, which increases the overall distance between loop and linker up to about 4 Å. The ATP to ADP exchange also affects hinge 225 separating domains IIA and IIB, which undergoes local unfolding in all trajectories, as well as strand 214–222, that partially loses the β-like coordination to strand 197–204. The apo system populates a conformation characterized by an enlarged binding pocket, induced by the rigid rotation of subdomain IIB around hinge 308 relative to IIA, coupled to a partial helix unwinding at hinges 308 and 225. This rearrangement is not observed in either ligand-bound state.

The distance fluctuations (Figure 4) show comparable values to the ones obtained for the closed conformation. The analysis of NBD highlights marked differences in the overall internal coordination of ATP-bound and ADP-bound states with respect to the apo system. On average, the latter shows relative mobility of lobes I and II around loop 180, a highly mobile hinge around residue 300, indicating intrinsic conformational freedom of subdomain IIB with respect to IIA, and low coordination with SBD (Fig. S3). The ATP-state does not show significant subdomain motions (Figure 4, S4 B) and in particular the surrounding of the linker region around loop 210 appears rather rigid. In contrast, ADP strongly enhances the mobility of subdomain IIB and partly IIA (residues 230 to 360) with respect to the adjacent subdomain IA. Therefore, the most significant effect of ADP on NBD in the *open* state of DnaK is again to increase the mobility of lobe II with respect to lobe I.

The specific nucleotide in the NBD of DnaK encodes a conformational signal that is transmitted to the SBD, as demonstrated by the structural rearrangements. From the dynamical point of view, the map of distance fluctuations (Figure 4, S4) shows the same ligand induced modulation already seen in the *closed* DnaK structure. The βSBD is internally more rigid when ADP is bound than when ATP is bound, which is compatible with the structural finding of a rigid-like rotation of the subdomain.

#### The non-allosteric homolog Hsp110

The above described analyses are also applied to yeast Sse1, the non-allosteric homolog of Hsp70. In spite of the similarity with the modeled *open* DnaK presented above, the conformational evolution of *open* Sse1 is rather limited in the presence of all ligands. Overall, the binding pocket appears much less flexible and less modulated by the ligand than what can be observed for DnaK. The helix containing D175 (E168 in DnaK), as well as the two β-hairpin clamps (loop 195 and loop 10) (Figure 3) are stably oriented towards the nucleotide. Loop 210 is maintained close to the starting structure. The sequence stretch 187–196, corresponding to loop 180 in DnaK, but longer and enriched in proline residues, is rigid in the initial conformation and shields the adjacent loop 210 from solvent. The latter is shorter than in DnaK and stably coordinated with the linker in all ligand states. This result markedly differs both from the ATP-induced structural modulation observed at the linker in *closed* DnaK in presence of ATP and, more importantly, from the rearrangement occurring in the *open* DnaK in presence of ADP, despite the latter structure is homology modeled upon Sse1.

Although no significant ligand dependent structural changes are found in the NBD of Sse1, a ligand dependent modulation of conformational dynamics involves helix 232–252 (225–245 in DnaK) of subdomain IIB and, on the opposite side of the gate leading to the nucleotide binding site, residues 80–100 (82–101 in DnaK) of subdomain IB. The former helix is significantly bent at residue 243 (239 in DnaK) in the free system, and partially also in the ADP-bound system, whereas it conserves the original shape in the ATP-state. The dynamical analysis (Figure 4) again confirms the increased mobility of subdomain IIB in the absence of ATP, as already found in DnaK, due to the absence of coordination with the nucleotide terminal phosphate group. In contrast to DnaK, and in agreement with the structural findings, the inter-lobe coordination between I and II is maintained in all states and the linker-bound hydrophobic cleft around loop 210 does not appear to be ligand-modulated.

Finally, consistently with what observed above, the representative SBD structures of the most populated clusters (Table S1) in all ligand states are characterized by a constant orientation of the βSBD, showing a ligand independent behavior.

### Molecular basis of the communication between nucleotide and substrate binding domains

In the previous sections we identified significant differences in the global structure and dynamics of DnaK and Sse1 in response to ATP or ADP binding. In particular, we observed that the non-allosteric protein is not modulated by the ligand exchange at the lobe I-lobe II interface. In this section we aim to gain residue-based insight into the structural and dynamical rearrangement leading to the allosteric signal between the nucleotide binding site, the linker region and the SBD in the different ligand states of DnaK and compare them to Sse1. The residue-based modulation is analyzed by considering the time-dependent dynamical evolution of geometric strain and the average conformational mobility to identify mechanical hinges. Geometric strain is a measure of the time-dependent local deformation of the structure with respect to the average conformation (see Materials and Methods for details). Although not measuring the energy involved in the deformation, this quantity monitors protein areas undergoing significant microscopic rearrangements. Namely, regions involved in conformational changes show strain peaks, which can be related to local structural changes during the structural transition in response to a nucleotide.

To complement the dynamical analysis, the network of ligand-modulated hydrogen bonds connecting nucleotide binding site and SBD and supporting the allosteric communication is analyzed.

#### Geometric strain

In order to highlight the residues involved in conformational changes, we followed in detail the time-dependent geometric strain in two MD runs of DnaK where the most significant progress along the *open/closed* transition is observed in both directions (Figure 2, Video S1 and S2), i.e. ATP-bound *closed* DnaK (trajectory 3) and ADP-bound *open* DnaK (trajectory 1). The time-dependent strain analysis shows that, as ATP is bound to the closed conformation, the rotation of subdomain IIB is induced from the top of the helix connecting IIA and IIB, through the hinge at residue 305. Moreover, displacement of βSBD is anticipated by strain propagated through the linker, once the contact between IA and IIA has been enhanced by the rearrangement of loop 210. On the other hand, ADP binding to the open conformation induces the motion of subdomain IIB by increasing the mobility of the hinge residues 228 and 305. Also the segment 152–165 in subdomain IA, and particularly R164, whose mobility increases in the presence of ADP, is involved in the interaction with lobe II. Strain accumulation on residues at the αSBD interface and at R164 is followed by the displacement of αSBD towards the peptide cavity closure. Finally, in the SBD, residues 447–452 forming a loop in the βSBD are involved in the onset of nucleotide-induced changes in both directions, while residues 518–528 respond with the unfolding of helix AB. For more details, see Supporting Information.

#### Average conformational mobility

**Figure 5:**
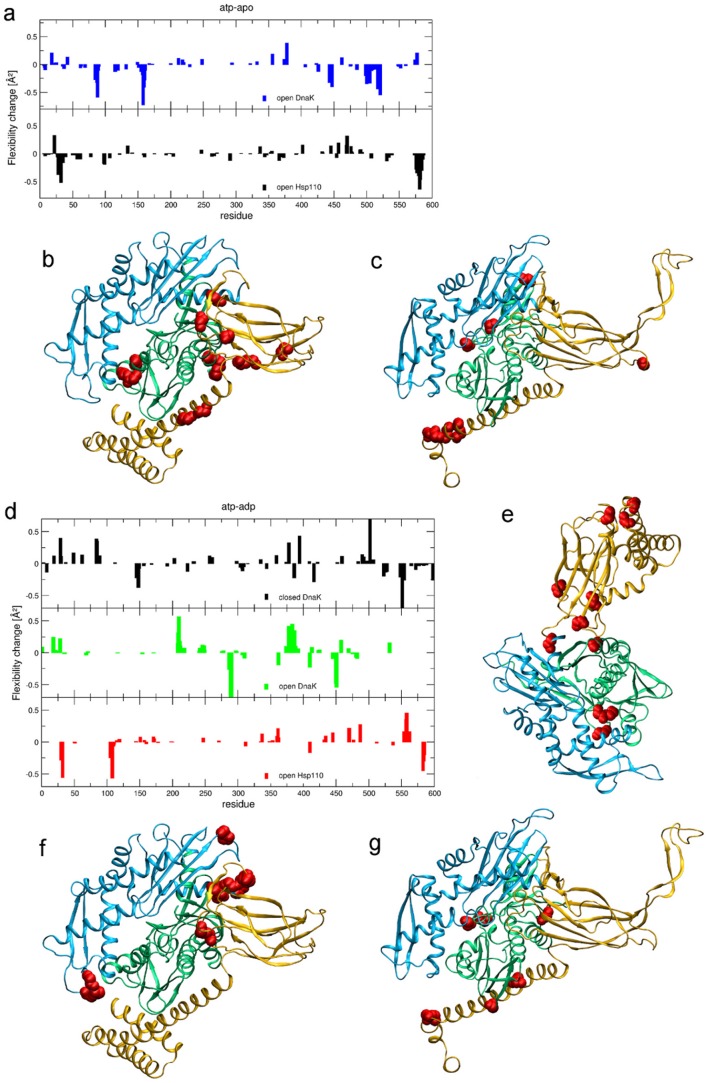
Average flexibility modulation. a) Histogram showing change in local flexibility of each residue going from the apo to the ATP bound state. Differences within statistical error are not considered. Top: *open* DnaK. Bottom: Sse1. b)–c) Flexibility change peaks in DnaK (b) and Sse1 (c) reported as spheres on the 3D structure. d) Histogram showing change in local flexibility of each residue going from the ADP to the ATP bound state. Differences within statistical error are not considered. Top: *closed* DnaK; middle: *open* DnaK; bottom: Sse1. e)–f)–g) Flexibility change peaks in *closed* DnaK (e), *open* DnaK (f) and Sse1 (g) reported as spheres on the 3D structure.

To complement these findings with an average, equilibrium measure of dynamical response, we analyze the conformational mobility, defined as average deformation that is locally experienced by each residue in a given nucleotide-bound state considering the whole set of trajectories. This is done by calculating the time-averaged geometric strain over each MD run, then by averaging the profile over the three MD runs of each system, to obtain the residue-based mobility (see Materials and Methods). By evaluating the mobility difference between the ATP and apo complexes of a given state (*open*-*closed*) and considering only statistically significant differences, a histogram is defined summarizing the dynamical modulation induced by ATP on the nanosecond scale. Comparing the *open* DnaK and Sse1 histograms (Figure 5a–c), the modulated hotspots are differently distributed on the structures. This is particularly interesting given that the two ATP complexes maintain a similar domain arrangement along the trajectory. In detail, ATP induces in DnaK a significant mobility increase at loop 210 and C-terminal helix-linker, and rigidity of segment 84–94 and 152–165. The Sse1 flexibility pattern is significantly modulated only around residue 30 in the NBD and no strong effect is found on regions involved in the NBD-SBD interface. As to the SBD, in presence of ATP residues around 580 become more rigid in Sse1, while in DnaK a significant coordination increase is found both around residue 450 and around residues 500–530, indicating the absence of conformational mobility in this area when ATP is bound.

To single out ligand specific effects, we also evaluate the local mobility difference between the ATP and ADP state of each system (Figure 5d–g). The increase in flexibility found at loop 210 and in the C-terminal residues 370–395 is specifically due to ATP both in the *open* and in the *closed* structure, while ADP in the *closed* DnaK structure induces mobility at residues 145–160 relative to ATP. An outstanding increase of mobility is evident at residue 502 in the *closed* DnaK bound to ATP, which acts as hinge for the opening of the SBD. Again, ligand-dependent modulation at the NBD-SBD interface is not found in Sse1, where spots displaying significant mobility increase upon exchange of ATP with ADP are mainly located at subdomain N-terminal NBD and in the terminal part of αSBD.

#### Hydrogen bond network

**Figure 6:**
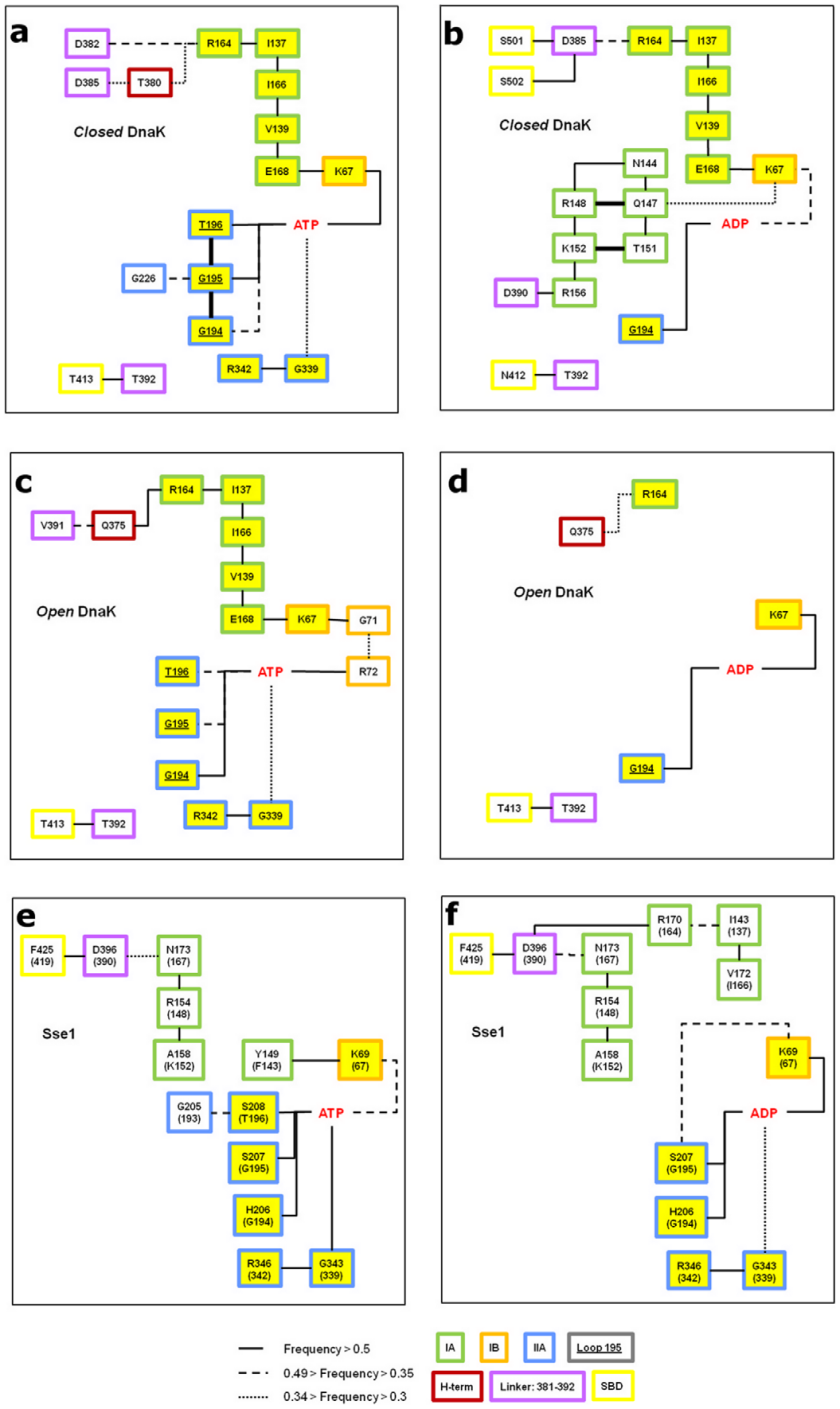
Hydrogen bond network. Map illustrating the persistent hydrogen bonds connecting nucleotide to NBD C-terminal helix and linker. Residues are color coded according to the subdomain they belong to. Yellow boxes refer to residues involved in conserved interactions network. Numbering refers to DnaK and connections are drawn based on the interaction lifetime. Left, ATP complexes (a, c, e); right, ADP complexes (b, d, f).

The aim of this analysis is to detect possible pathways underlying the propagation of the conformational signal encoded by the nucleotide through the structure [25]. We focus on the amino acid network connecting the nucleotide-binding site to the linker and to the SBD in DnaK and in Sse1 bound either to ADP or ATP. The presence of hydrogen bonds is detected along the simulation time of each single MD run. The persistence of identified bonds is then evaluated by considering the three trajectories together (see Table S2 for single trajectory details). Overall, the frequency of most populated hydrogen bonds is conserved among the three trajectories of the same complex. This leads us to defining a network, which is summarized in Figure 6. Interestingly, there is evidence of a ligand modulated hydrogen-bond network in DnaK, which is not found in Sse1. The main conserved hydrogen bond-based connection between the nucleotide site and the linker is found in all DnaK systems and consists of residues K67, E168, V139, I166, I137 and R164, which link the nucleotide to the NBD terminal helix. These residues are conserved across the Hsp70 family. Interestingly, this pathway appears more stable in the presence of ATP, due to the coordination between the phosphate group and K67 and E168. In particular, it is disrupted in the *open* DnaK bound to ADP, which undergoes the opening transition. None of the Sse1 systems retains this network.

Another interesting nucleotide-modulated interaction involves the conserved loop 195. In particular G194, G195 and T196 are involved in hydrogen bonds in the *open* and *closed* DnaK conformation bound to ATP. In presence of ADP the coordination to the loop is markedly reduced, only G194 remains connected to the nucleotide. Moreover, in Sse1 residues GGT are substituted with the less flexible HSS and the interaction is no longer affected by the nucleotide type. Another ATP-driven connection in DnaK is represented by the link to the conserved residue R342, mediated by G339, present in the hydrogen bonds network of *open* and *closed* system. This residue is localized at the interface between subdomain IIA and IIB and this interaction can be involved in the rotation of subdomain IIB, which determines the NBD opening of the ATP-bound state. These residues do not appear in the DnaK-ADP network. In contrast, Sse1 shows this connection in presence of both ligands, suggesting the absence of a ligand modulation on the IIB subdomain rotation. The *closed* DnaK-ADP complex presents a second hydrogen bonds network connecting K67 to D390. Interestingly, mutants of residue belonging to this network, R148 and K152, display a complete loss of interdomain communication [13]. Other two relevant residues, D385 and D390, are involved in the *closed* DnaK-ADP complex, while only D390 is included in the hydrogen bonds network of DnaK-ATP complex. These two residues flank the conserved hydrophobic VLLL sequence of the interdomain linker, which is involved in the allosteric coupling of the two domains [7].

A connection between the linker and the SBD, present in all DnaK simulations, is given by T393, contacting T413 or N412, depending on the nucleotide state. N412 is well conserved in the Hsp70 family with respect to the Hsp110. In general, most of the residues included in the identified DnaK hydrogen bonds network are highly conserved among Hsp70 family, whereas in the Hsp110 these positions are often substituted (Table 1).

**Table 1:** Conservation of Hsp70 and Hsp110 residues involved in the hydrogen bonds network.

Residue	Subdomain	Hsp70 variants	Hsp110 variants
**K67**	IB		**R**
I137	IA	V	L/M
V139	IA	C	V
N144	IA	D/T	G/K
Q147	IA	E	E
**R148**	IA		
T151	IA		L/A/M/V
**K152**	IA	**R/Q**	**L/A/M/I/V**
R/V156	IA	T/A/E/K/Q	N/D/E/Q/K
**R164**	IA		G
I166	IA	V	V/M
**E168**	IA		**D/A**
G194	IIA		
**G195**	IIA		**H/S/F**
**T196**	IIA		**S/G**
G339	IIA		
R342	IIA		I
**D385**	linker	**E/G**	**E/P/K/R**
**D390**	linker		
T392	linker	A/N/V	N/V
N412	βSBD		S/P/C/K
T413	βSBD	S/A	E/K/R

In bold residues having known mutants that present impaired function.

**Figure 7:**
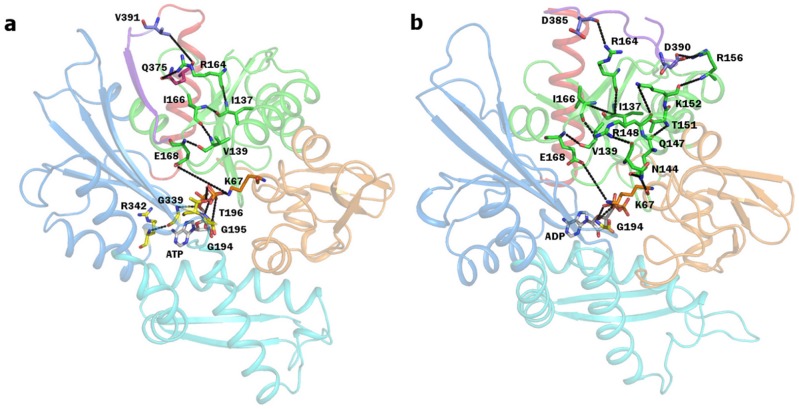
Hydrogen bond network. Structural representation of the persistent hydrogen network connecting nucleotide and linker as observed in the MD trajectories in a) ATP bound *closed* DnaK; b) ADP bound *closed* DnaK. Residues and nucleotides are shown in stick model and colored by atom type, carbons are colored based on subdomain.

Overall, by means of this analysis we have been able to identify two significant groups of interactions that are specific of DnaK (Figure 7), respond to the bound ligand and connect the nucleotide binding site to the SBD. Both of them appear to be stabilized by ATP and are not conserved in Sse1. One such network extends from the binding site to the terminal helix through subdomain IA starting from the catalytic residues K67 and E168, while the other, belonging to subdomain IIA, coordinates the nucleotide phosphate to loop 210, via loop 195.

### ENM-based model of the open-close transition

The all-atom picture of the conformational transition between open and closed state of DnaK in the presence of ADP, and in the opposite direction in the presence of ATP, presented in the previous sections, consists of two subsequent conformational events occurring in a well-defined order: namely, in the closing transition one has detachment/local unfolding of αSBD, followed by the displacement of βSBD, while in the opening transition the two steps are reversed (first step is onset of docking of βSBD mediated by the loop 210-linker interaction, followed by unfolding at αSBD). The conformational changes from open to closed structure or vice-versa are compared to those obtained with a coarse grained approach based on Elastic Network Models. By imposing the known DnaK start and end conformation and using the PATH-ENM tool from the AD-ENM Web Server [26], we simulate a transition pathway connecting the two states, based on the most representative normal modes of each conformation. Interestingly, the order of events observed in both directions corresponds to what suggested by our all-atom approach. See Supplemental Information for details (Figure S7).

On the other hand, the correspondence between all-atom and coarse-grained approach is lost when comparing the internal dynamics of Sse1 and of the homology modeled open DnaK. The three most relevant normal modes of the two proteins, calculated by means of the AD-ENM server [26] show a significant similarity (Figure S8) as expected because of the structural homology between the two proteins. Also, the prediction of hinges by means of Anisotropic Network Model (ANM) web server HingeProt [27], [28] locates hinge residues at essentially the same positions (393, 520, 510, 537, DnaK numbering) in all cases. Therefore, no indication of differentiated ligand-based modulation of the dynamics of Sse1 and DnaK can be obtained by applying ENM-based methods to these systems, despite the fact that the coarse-grained methods catch the global displacements highlighted by the all atom analyses.

## Discussion

The aim of this study was to investigate by MD simulations the molecular basis of allosteric communication mechanisms in DnaK in contrast with the non-allosteric behavior of Sse1. The conformational transition induced by ATP on the *closed* state of DnaK, that is the linker docking to the hydrophobic binding pocket at the NBD and the αSBD opening, consists of significant subdomain rearrangements. Computational investigations of large-scale structural changes usually rely on coarse-grained models, which, by making use of simplified protein representations and, in some cases, of the knowledge of the initial and final states of the transition, can efficiently model sizeable rearrangements and provide useful information on the underlying mechanisms.

Elastic Network Models can be used to retrieve collective, functionally relevant motions [27], [28], [29] around the specific structures on which the Hamiltonian of the system is built and allow to predict possible transition pathways between two given conformations, based on native fluctuations. Recently, a coarse-grained approach based on UNRES MD simulations [22] was used to model the full conformational transition in Hsp70, by posing distance constraints on the NBD subdomains that simulate the presence or absence of bound ATP. The motion of SBD and NBD domains was shown to be modulated and, interestingly, the occupancy of open and closed SBD states was found to correlate with the “ligand” presence, in agreement with experimental data. Overall, coarse-grained methods can shed light on ligand-activated modulation of protein motions, since the latter are largely determined by the structural organization of the native state. However, such models lack by definition the atomic detail that is required to understand the finely tuned physico chemical origin underlying ligand-based modulation. Similarly, differences arising from sequence divergence in homologues, such as the ones observed between Hsp70/DnaK and Hsp110/Sse1, cannot easily accounted for by coarse-grained methods. The question addressed in the present work, namely to elucidate the molecular mechanism underlying Hsp70 allostery in comparison to its non allosteric homologue Hsp110, can therefore take advantage of full atomic detail, as shown in the previous section in comparison to ENM results. On the other hand, a well-known limitation of all-atom MD simulations is sampling. Large conformational rearrangements may be out of reach for a single 100 ns MD simulation run. Enhanced sampling methods like accelerated MD [30], as well as non-dynamical energy optimization pathways strategies [31], [32] can provide a high resolution model for a complex structural transition with higher efficiency than unbiased MD. As an alternative, information on the global conformational changes may be inferred from standard MD trajectories by extrapolating collective motions inducing the transition by means of PCA methods. Such an approach was recently attempted for Hsp70 by Nicolai et al. [21] with the aim of defining the dynamic modes involved in the transition. With a complementary point of view, in this paper we have applied a set of recently developed methods [15], [16], [17], [18] that allow us to identify the relevant residues which are involved in the early onset of a conformational change in DnaK. We comparatively analyze the structural and dynamical changes that occur at the single residue level on the ns scale and relate these to the complete conformational transition. The underlying reasoning is that transitions can be triggered or favored by networks of interconnected residues that respond to specific signals (ligand binding, exchange or even covalent modifications) by changing their dynamic states. The link between residue-level changes in protein dynamics and long-range propagation of allosteric signals has been probed by NMR analysis [33], [34], [35]. Therefore, even if we are still unable to observe full conformational changes, the theoretical identification, coupled to validation against experimental data, of functionally relevant residues throughout the structure of the protein, in explicitly distinct ligand states, helps to shed light on the molecular determinants of allostery in different proteins of the same family. In our study, while the progress of the opening transition in ATP-bound *closed* DnaK is observed only at an initial stage, the onset of the opposite closing transition occurring in the modeled *open* DnaK, with the αSBD detaching from the NBD domain, is detected in our MD trajectories to a significant extent. In both directions the transition is the result of consistent microscopic modifications, such as spatial rearrangements or dynamical modulation of specific residues, which work as rigid units and flexible hinges and respond to the specific ligand. The comparison between DnaK and Sse1, where such conformational changes are not observed, helps validate our observations and provides a model for the allosteric mechanism in Hsp70, identifying relevant structural residue hotspots at the atomic level.

Our combined analysis identifies two pathways transmitting the ligand encoded signal: loop 195, interacting with ATP, appears as the most relevant sensor and induces a dynamical modulation at loop 210. In parallel, the coordination with K67 and E168 stabilizes the hydrogen bond network that connects the binding site, through domain IA, to the C-terminal residues and the linker. The combined effect of such interactions results in the stiffening of the interface between lobe I and lobe II and induces the conformational rearrangement of loop 210. The stabilization of the interface between lobes I and II and with the linker is in turn reflected by the increased coordination of the βSBD with the NBD. In the presence of ADP, the increased linker mobility, due to the loose coordination between the nucleotide and C-terminal end of NBD, as well as the relative mobility of lobes IA and IIA, stimulates motion through a hinge located at residue D390 that is propagated to the βSBD and sets up its rigid movement. Interestingly, the latter is coupled to an increased mobility of NBD subdomain IIB.

This picture is supported by the dynamical and sequence-based comparison with the non-allosteric Sse1. In particular, loop 195 and loop 210 shows a different sequence composition in Sse1. This is likely to induce a different dynamical behavior, as pointed out in the Results section. In Sse1 the persistence of interactions between loop 195 and nucleotide after ATP-ADP exchange, as well as the increased solvent protection of loop 210 through the adjacent rigid loop 180 traps the structure of the interface between domains IA and IIA of the ATP state in a stable conformation.

Available mutational data confirm the relevance of the interactions between loop 195 and nucleotide. Mutants of G195 disrupt the ATP-induced structural dynamics [36], while T196 mutants have a reduced ATPase activity [36]. Also, the network originating from K67 is known to be essential for ATPase activity and for inter-domain communication, and mutants of this position display a reduced ATPase activity [37]. The same holds for E168 mutants [36], [37] which induce impairment of interdomain communication [13], [36], uncoupling ATP activity and substrate release.

Recent NMR work [10] has identified the protein hotspots, constituting the allosteric network in NBD, that respond to the nucleotide exchange with a conformational transition by means of chemical shift perturbation. The identified network consists of the linker and the IIA β-sheet connecting loop 210 and loop 195, which is in agreement with our simulations results. Interestingly, our flexibility analysis investigates motions on the ns-scale, when conformational changes have not completely occurred yet. It is worth noting that the relative flexibility increase at loop 210 and at the linker in presence of ATP compared to ADP point towards an activation of the same region. Our dynamical results can also be qualitatively compared to hydrogen exchange data, reporting on general flexibility properties [38]. Regions that undergo a modulation of protection to hydrogen exchange, in particular NBD linker and SBD residues 400–500, are affected in our simulation by a significant change in the dynamical pattern that may anticipate the structural rearrangements. Flexibility changes upon nucleotide exchange, in the non-allosteric Sse1, are differently distributed on the protein structure, do not involve the interface between NBD and SBD and are in general less intense. These observations support the relevance of the dynamical modulation to the Hsp70 allostery.

The binding site of the ATPase-stimulating cochaperone DnaJ has recently been reported to be highly dynamical and located along the β-strand 220 [39]. DnaJ is hypothesized to bind to the *open* Hsp70 structure and enhance ATP hydrolysis, thereby favoring the substrate- and ADP-bound conformation. Considering the *open* DnaK state simulation in the presence of ATP, we observed increased rigidity along strand 220, which propagates to domain IIB. The bound cochaperone can enhance this rigidity and improve the coordination between lobes I and II, hence promoting ATP-hydrolysis. Moreover, the Gestwicki group identified small molecules that bind to the cleft between IA and IIA and work as stimulating effectors of ATP hydrolysis in synergism with DnaJ [40]. One of these compounds is found to bind to the same β-strand involved in DnaJ interaction, which further confirms the main functional role played by this protein region. According to our simulations, the coordination of domain IIA and IIB is modulated by the presence of ATP in both DnaK simulations. The motion of subdomain IIB with ADP is due to the enhanced mobility of strand 220 and of residues 308, as a consequence of the reduced interaction between loop 195 and nucleotide upon ATP-ADP exchange, inducing the opening of the binding clamp. The effect of the nucleotide exchange on the modulation of the subdomain arrangement in the NDB has been object of experimental and computational investigation. In particular, MD simulations [41] confirmed by NMR data on a homologue system [42], showed that nucleotide exchange AMPPNP-ADP induces a rotation of domain IIB coupled to the opening of the nucleotide-binding pocket. In agreement with these results, we observe that ADP significantly affects the mobility of IIB by increasing the flexibility of two hinges, residues 225 and 308, and hence opening the binding clamp. Moreover, a reduced connection of domain IIB is displayed also by the absence of interaction between ADP and residue R342 at the interface between domain IIA and IIB, in both conformations, *open* and *closed*. Mutational studies [36] show that residue substitutions around hinge 225 affect the ATPase rate, although they do not perturb the in vivo refolding function. Binding of small molecules in the vicinity of this region in the ADP state and not in the ATP state strongly suggest a ligand-induced modulation of the binding area [43].

The consistency between our dynamical data and experimental results suggests that the dynamical effect induced by ligands on MD-compatible time scales can shed light on the molecular basis of allosteric mechanism coupled to large structural rearrangements. A systematic comparison between Hsp70 and its non-allosteric homologue Hsp110 to single out sequence determinants of Hsp70 allostery, by means of Statistical Coupling Analysis (SCA), was recently performed [19]. A sparse network of coevolving residues that differentiate Hsp70 and Hsp110 families has been identified and hence these residues have been suggested to be necessary for determining the allosteric mechanism in Hsp70. We have focused instead on the mechanistic differences between DnaK and Sse1 with an all-atom approach and have identified those protein segments that display a different dynamical behavior in the two proteins, either increasing the coordination between nucleotide binding site and SBD or acting as flexible hinges. The hotspots we identified, comprising the β-strands 220, loop 195, loop 210 and 180 in lobe II and the hydrogen bond network connecting nucleotide to the linker, provide the minimal mechanistic unit that can be considered allosterically responsive. The comparison with the set of residues provided by Smock et al. returns a common group of residues, including E168, loop 195, T151, K152, N412 and T413. As expected due to the differences in the nature of the analysis, the two sets do not fully overlap, since some amino acids involved in the allosteric mechanism may be conserved between Hsp110 and Hsp70. Also, not every residue of the SCA sector is expected to be directly essential for the allosteric function per se, but might be relevant to ensure stability and compensate for other functionally relevant mutations. In contrast to the SCA analysis, no strongly responsive regions were identified on the β-sheet body of βSBD, but only on its loop regions. This discrepancy might also be influenced by the absence of a peptide substrate in our simulations.

Overall, by crossing the dynamical and the sequence information, a subset of critical positions affecting allosteric communication can be promptly identified and rationalized in a mechanistic perspective. By complementing sequence and structural-dynamical analysis one could hence define and explain the minimal required set of mutations that abolish allostery in Hsp70. More generally, by applying our dynamical analysis approach in a comparative study of an allosteric and a non-allosteric system, we demonstrated that including functional dynamics, internal residue-residue coordination, and protein flexibility information, could help unveil ligand-responsive regions and possible binding sites of a protein with allosteric properties, which may not be immediately evident in a single-structure representation. This offers the opportunity of modulating protein function by specifically addressing regions crucial for the functional dynamics of the protein through specific mutations or small molecules targeting allosteric sites different from the classical binding site targets.

## Materials and Methods

### Protein structures

As initial structure of DnaK, an Hsp70 homolog, the X-ray structure of E. coli DnaK (PDB ID: 2KHO [9]) in complex with ADP-Mg^2+^ was employed. ATP complex was built by substituting ADP with ATP-Mg^2+^ coordinates obtained from 2EA8 structure [44]. Hsp110 X-ray structure of S. cerevisiae (PDB ID: 3C7N_A [12]) in complex with ATP-Mg^2+^ was utilized as starting point. For the ADP complex, ligand coordinates were substituted with ADP-Mg^2+^ obtained from 1S3X [45]. The apo forms were obtained removing ligand coordinates. The DnaK *open* conformation homology model, bound to ATP, was obtained from a previous study [19]. ADP complexes and apo structure were built as previously described.

All complexes were solvated in a triclinic box of SPC water keeping a minimum distance of 1 nm between the solute and each face of the box. This results in about 100.000 water molecules included in the DnaK and Sse1. Total charge was neutralized with Na^+^ ions added to the simulation box at random positions.

### Molecular dynamics simulations

Molecular dynamics simulations were performed with Gromacs 4.0 package [46], employing the GROMOS96 (ff43a1) force field [47]. All complexes were energy relaxed with 1000 step of *steepest-descent* energy minimization. MD simulations were performed using the LINCS algorithm [48] to constrain bond lengths and periodic boundary conditions were applied in all directions. Long-range electrostatic forces were treated using the Fast Particle-Mesh Ewald method (PME) [49]. Van der Waals forces and Coulomb potential were treated using a cut-off of 0.9 nm and the simulation time step was set to 2 fs. An initial velocity obtained according to a Maxwell distribution at 300 K was given to all the atoms. All simulations were run in NVT environment employing V-rescale as temperature coupling algorithm, with reference temperature set at 300 K.

Three independent simulations were run for both DnaK and Sse1. The total simulation time was 200 ns for ADP and apo states. Production runs for ATP complexes were extended to 225 ns and 210 ns for DnaK and Sse1, respectively.

### Molecular dynamics analyses

To evaluate the effects of ligand bound on single residues and on protein domains and the intrinsic differences between DnaK and Sse1, different analyses were carried out on the equilibrated trajectories.

#### Clustering

Cluster analysis was obtained using *g_cluster* module of Gromacs to evaluate the long-range effects of bounded ligand on NBD and SBD. Clustering of the trajectories was carried out with gromos method [50] fitting Cα atoms of NDB or SBD with a cut-off of 0.5 nm and fitting on nucleotide binding site (residues 2–15; 160–225; 360–390) with a cut-off of 0.2 nm.

#### Distance fluctuations matrix

For the dynamical analyses, each MD trajectory was projected on the first 10 principal components [51] obtained by Essential Dynamics analysis [52] of the original MD run. Gromacs module *g_covar* and *g_anaeig* were employed to calculate the essential dynamics for each simulation. Each frame of the trajectory was aligned on the Cα atoms of the starting conformation and the covariance matrix was calculated on Cα atoms.

On the filtered trajectory we computed the matrix of distance fluctuations *A*:
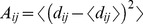
(1)where *dij* is the (time-dependent) distance of the Cα atoms of amino acids *i* and *j* and the brackets indicate the time-average over the trajectory. The *A* matrix, and various quantities derived from it, can be used to characterize the salient elasticity and plasticity properties of a protein undergoing structural fluctuations.

#### Geometric strain

By evaluating the time-dependent geometric strain profile, hinge regions for the quasi-rigid domain motion can be identified [53], consistently with the above observation. The strain of a given amino acid, *i*, is defined as
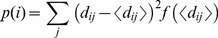
(2)where *j* runs over all protein amino acids, 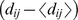 is the instantaneous deviation from the average of the distance between residue i and residue j, and *f* is a sigmoidal function that restricts the contribution to the sum to amino acids that are within about 7 Å from amino acid *i*: 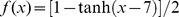. Hinge residues will be characterized by high values of *p*, because their local network of contacts appreciably changes due to the relative motion of neighboring residues, in the course of the dynamical evolution.

#### Conformational mobility

The conformational mobility per residue is obtained by averaging the geometric strain over the simulation time in each trajectory run:
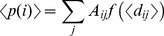
(3)


#### Hydrogen bond network reconstruction

*g_hbond* module of Gromacs, applied on the single trajectory, and in-house developed scripts have been employed to focus on the connection between nucleotide binding cavity and the SBD. For identifying the network of interaction involved in the signal transmission from the nucleotide to the SBD, the list of residues interacting with the nucleotide was obtained at first analyzing the trajectories with *g_hbond*. Each residue establishing a hydrogen bond with the nucleotide for at least 30% of the whole simulation time, considering the three trajectories, was subjected to a second stage of *g_hbond* analysis. From that, a new list of residues was obtained as in the first step, considering only residues forming H-bonds for at least 30% of the whole simulation time. This procedure was iterated moving from residues that form contacts with the ligand toward the linker (DnaK: residue 382–397; Sse1: residue 386–398) and an interaction network, involved in the signal transmission, was finally identified.

## Supporting Information

Figure S1 **RMSD of NBD (A, C) and SBD (B, D) of** ***open*** **,** ***closed*** **DnaK and Sse1 Bound to ADP (A, B) or ATP (C, D).** RMSD of the single trajectories, calculated fitting the backbone atoms. In red, orange and yellow the *closed* DnaK trajectories; in blue, light blue and green the *open* DnaK trajectories; and in violet, magenta and dark violet the Sse1 trajectories.
(TIFF)

Figure S2 **Secondary structure comparison.** Secondary structure composition of 100 conformations selected from the *closed* DnaK, *open* DnaK and Sse1 apo form trajectories.
(TIFF)

Figure S3 **Map of distance fluctuations of apo complexes.** Matrix of distance fluctuations calculated and averaged over the three MD trajectories for each apo complex. Matrix entries are colored black if below 0.3 and white if above 0.3 Å^2^, to highlight differences in rigidity pattern among the different complexes. Top, *closed* DnaK; middle, *open* DnaK; bottom, *open* Sse1.
(TIFF)

Figure S4 **Map of distance fluctuations of** ***closed*** **DnaK (A),** ***open*** **DnaK (B) and Sse1 (C) complexes.** Matrix of distance fluctuations calculated for the three MD trajectories for each ATP, ADP and apo complex. Matrix entries are colored black if below 0.3 and white if above 0.3 Å^2^, to highlight differences in rigidity pattern among the different complexes.
(TIFF)

Figure S5 **SBD opening in** ***closed*** **-ATP complex.** Representative cluster conformations of *closed* DnaK SBD bound to ADP (red) and to ATP (green).
(TIFF)

Figure S6 **RMSD map.** Map of time dependent RMSD comparison (snapshot to snapshot RMSD calculated on proteins Cα) between the III MD run starting from ATP-bound *closed* DnaK complex and the I MD run starting from ADP-bound *open* DnaK complex, showing an RMSD decrease towards the end of the trajectory, hence a convergent MD evolution to intermediate, semi-open similar structures.
(TIFF)

Figure S7 **Transition pathway connecting the two DnaK** ***open*** **and** ***closed*** **states.** Snapshots are extracted from the steepest-descent paths connecting *open* and *closed* DnaK structures, as obtained by applying the AD-ENM Web server (see main text). NBD lobe I is colored in green, lobe II is in light blue, βSBD is shown in yellow and αSBD is in red.
(TIFF)

Figure S8 **Normal mode analysis.** ENM-based analysis of homology modeled *open* DnaK (here referred to as Hsp70) and of *open* Sse1 (Hsp110). By superimposing extreme structures as obtained by AD-ENM Web server the motion along the first three normal modes (first, blue; second, grey; third, red) is shown (see main text). The sequence stretch 500–527 in Hsp110 was removed from the analysis to improve comparison.
(TIFF)

Table S1 **Trajectory clustering results.** The number of clusters obtained fitting on Total, NBD or SBD residues is specified in brackets, the percentage represents the number of conformations belonging to the most populated cluster.
(DOCX)

Table S2 **Persistence of the most populated hydrogen bonds during the single trajectories.** Percentage of presence, during the single simulation, for each hydrogen bond involved in the identified network.
(DOCX)

Video S1 **Geometric strain analysis of** ***closed*** **DnaK bound to ATP.** In the presence of ATP, the closed structure initially shows accumulation of strain at loop 210 in subdomain IIA and segment 152–165 in subdomain IA, which relaxes when lobe I and II become more tightly in contact. The rearrangement of segment 152–165 induces interaction and strain increase at the C-terminal end of the NBD terminal helix (D382 and V383), which act as hinge and induce the motion of the βSBD. Furthermore, the rearrangement of subdomain IIB is stimulated through strain increase at residue V328, at the beginning of the helix connecting subdomain IIA and IIB. Finally, a significant strain accumulation is observed at different spots in the SBD domain, such as the external loop 400 and loop 440, and residue 502, which acts as hinge for the SBD. Strain increase around residues 514–520 suggests local unfolding and the onset of the helix opening transition.
(MP4)

Video S2 **Geometric strain analysis of** ***open*** **DnaK bound to ADP.** In the *open* DnaK bound to ADP, a significant strain accumulation indicates approaching between residues of loop 220–224, and the opposite loop area of βSBD, residues 447–452, that progressively rearranges its contact with the NBD. Strain increases at hinges at the C-terminal end, D385, which is propagated through linker and D393 to the SBD, inducing the motion of βSBD. This event is followed by strain accumulation and release at the interface between NBD subdomain IB and the contacting αSBD (NBD residue 164 and SBD residues 505, 517, 522 and 525). As a consequence, segment 518–528, detaches from the NBD. Interestingly, in the presence of ADP strain increases at loop 195.
(MP4)
