# Supplementary figures and images for: Molecular Mechanism of Allosteric Communication in Hsp70 Revealed by Molecular Dynamics Simulations
Source: PLoS Comput Biol. 2012 Dec 27;8(12):e1002844. doi: 10.1371/journal.pcbi.1002844 (PMC3531320; doi:10.1371/journal.pcbi.1002844)

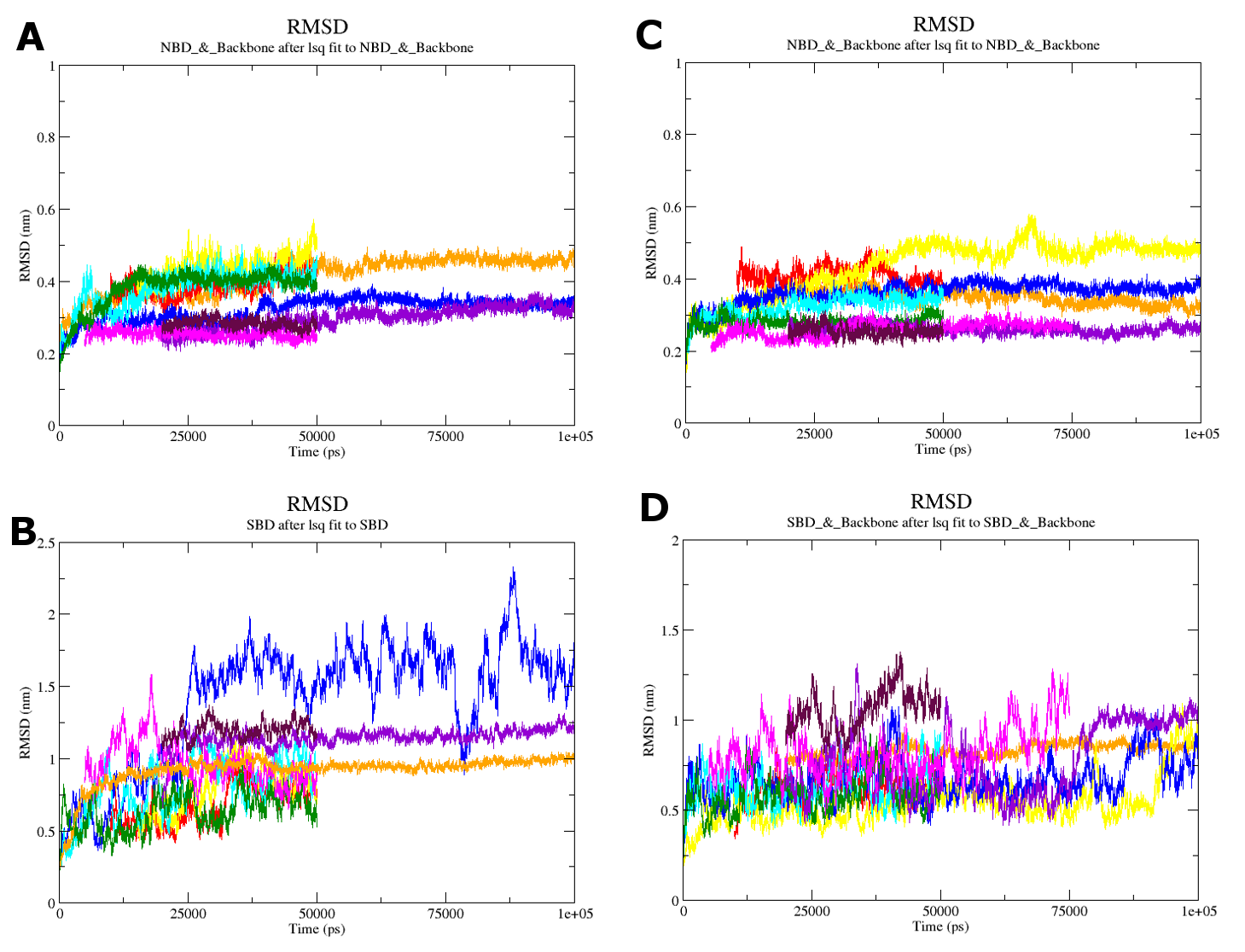

Supplement: Figure S1 — RMSD of NBD (A, C) and SBD (B, D) of open , closed DnaK and Sse1 Bound to ADP (A, B) or ATP (C, D). RMSD of the single trajectories, calculated fitting the backbone atoms. In red, orange and yellow the closed DnaK trajectories; in blue, light blue and green the open DnaK trajectories; and in violet, magenta and dark violet the Sse1 trajectories. (TIFF) [file pcbi.1002844.s001.tiff]

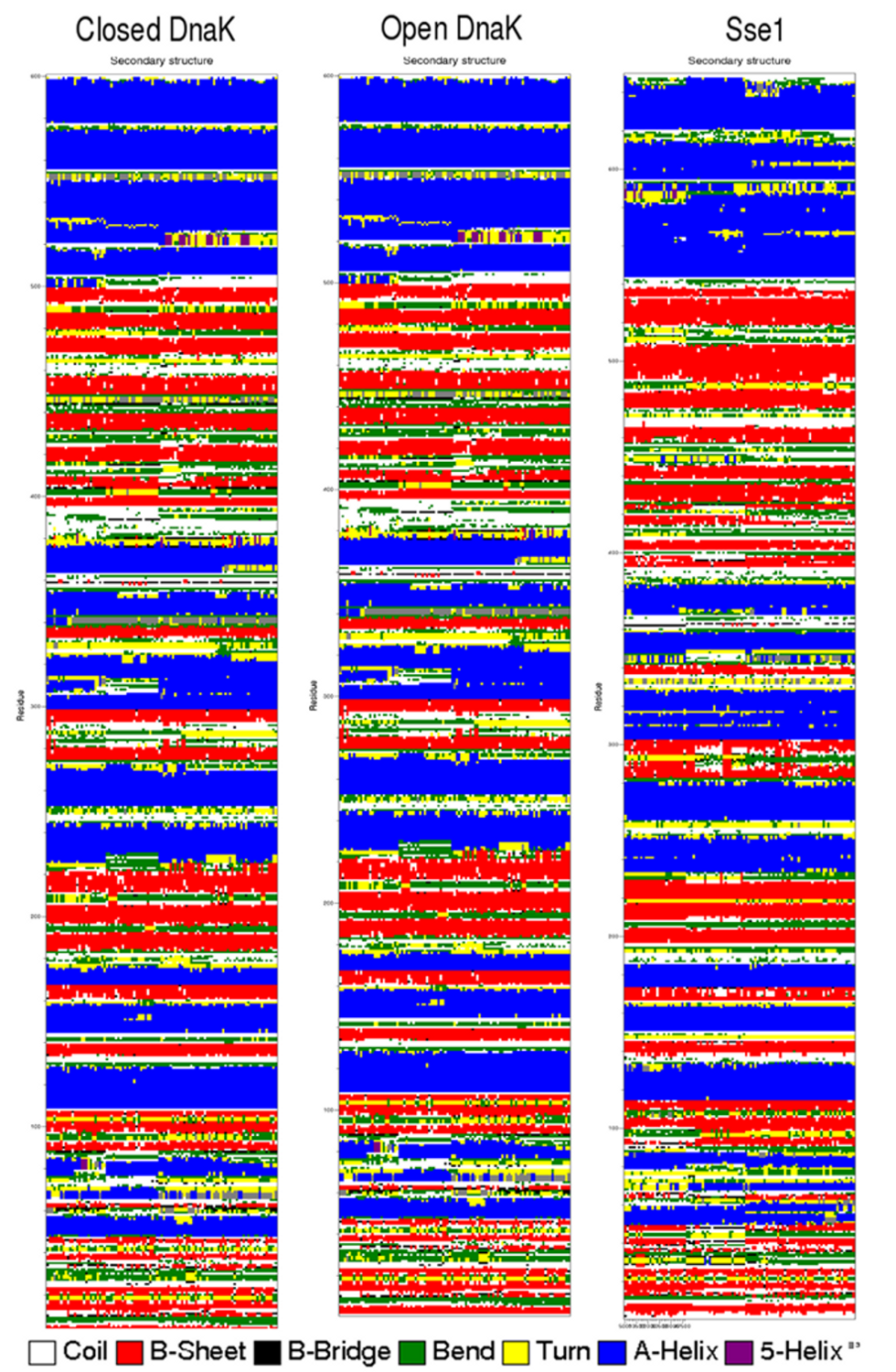

Supplement: Figure S2 — Secondary structure comparison. Secondary structure composition of 100 conformations selected from the closed DnaK, open DnaK and Sse1 apo form trajectories. (TIFF) [file pcbi.1002844.s002.tiff]

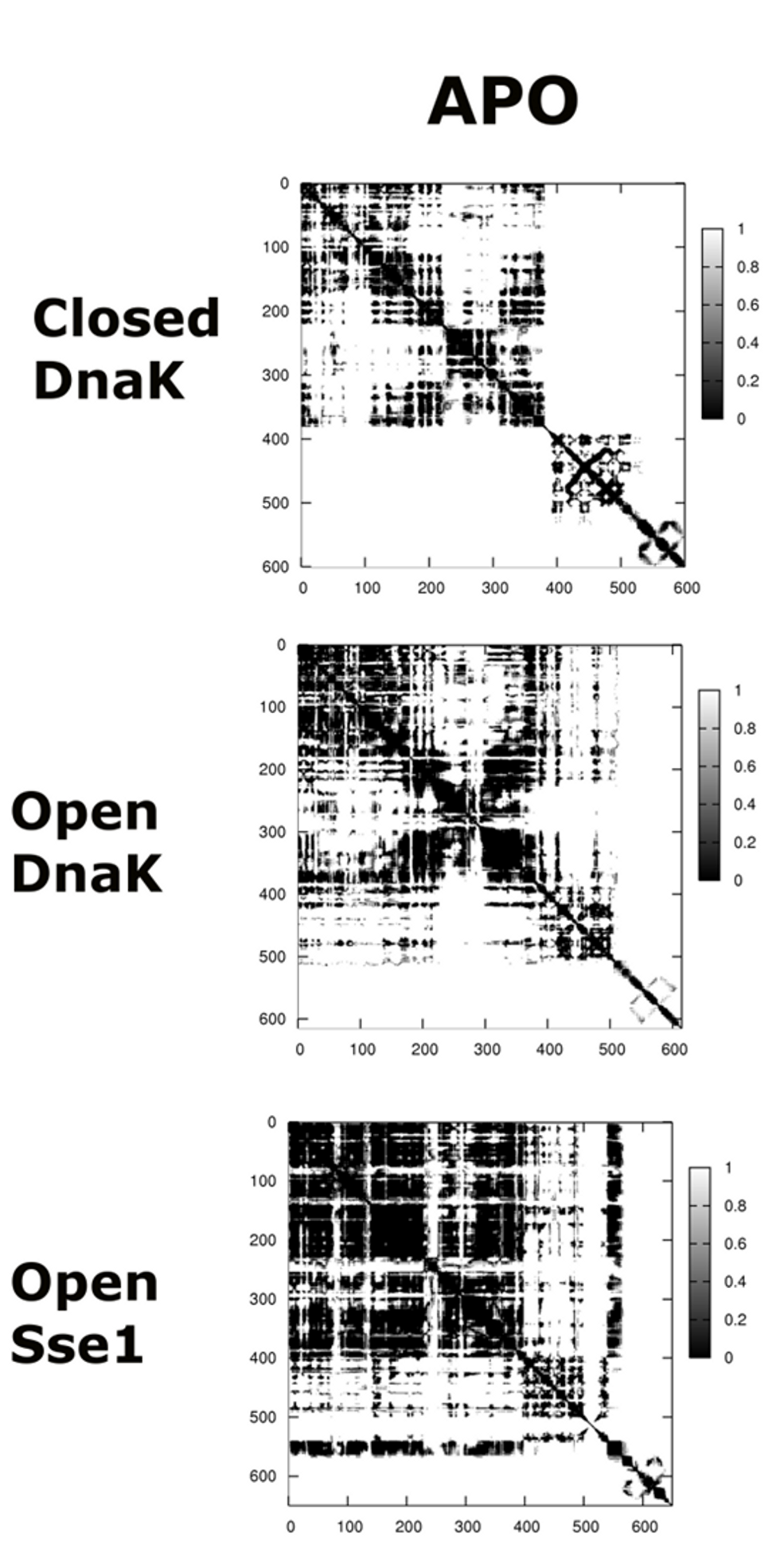

Supplement: Figure S3 — Map of distance fluctuations of apo complexes. Matrix of distance fluctuations calculated and averaged over the three MD trajectories for each apo complex. Matrix entries are colored black if below 0.3 and white if above 0.3 Å2, to highlight differences in rigidity pattern among the different complexes. Top, closed DnaK; middle, open DnaK; bottom, open Sse1. (TIFF) [file pcbi.1002844.s003.tiff]

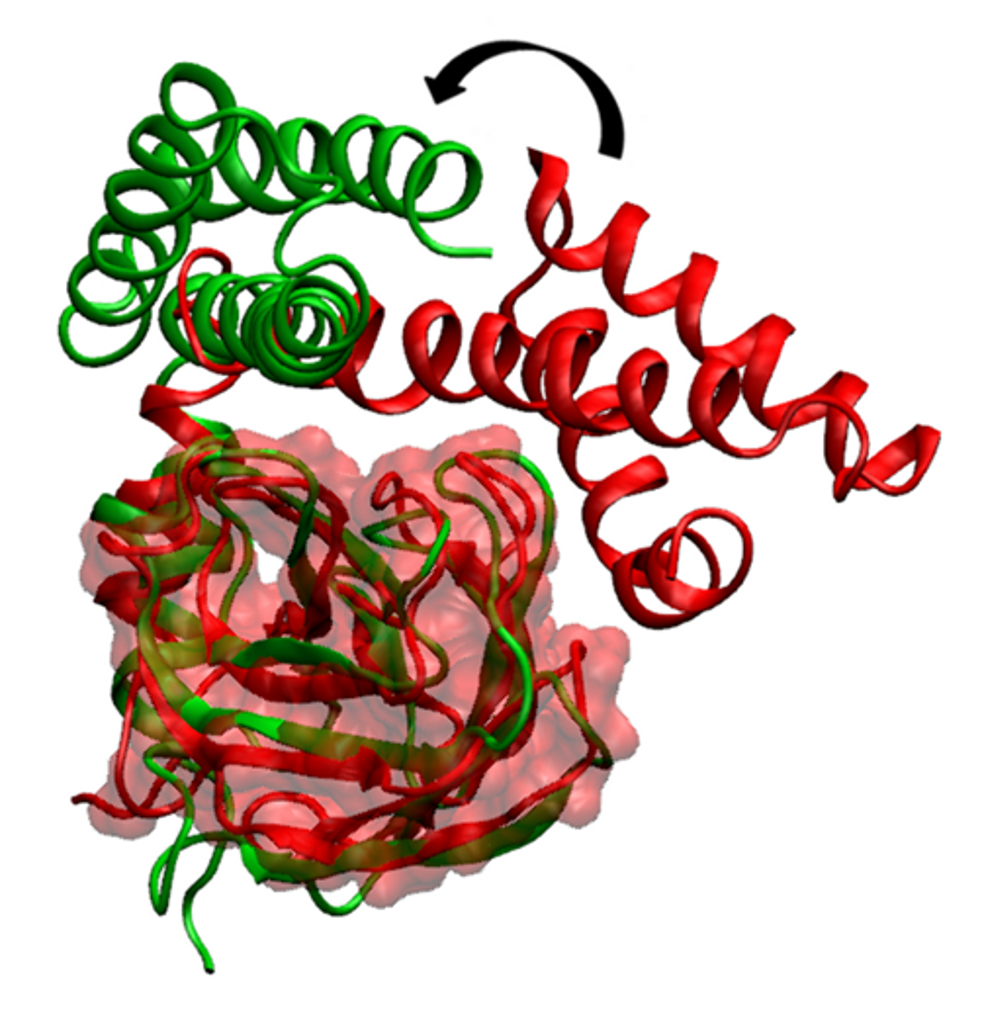

Supplement: Figure S5 — SBD opening in closed -ATP complex. Representative cluster conformations of closed DnaK SBD bound to ADP (red) and to ATP (green). (TIFF) [file pcbi.1002844.s005.tiff]

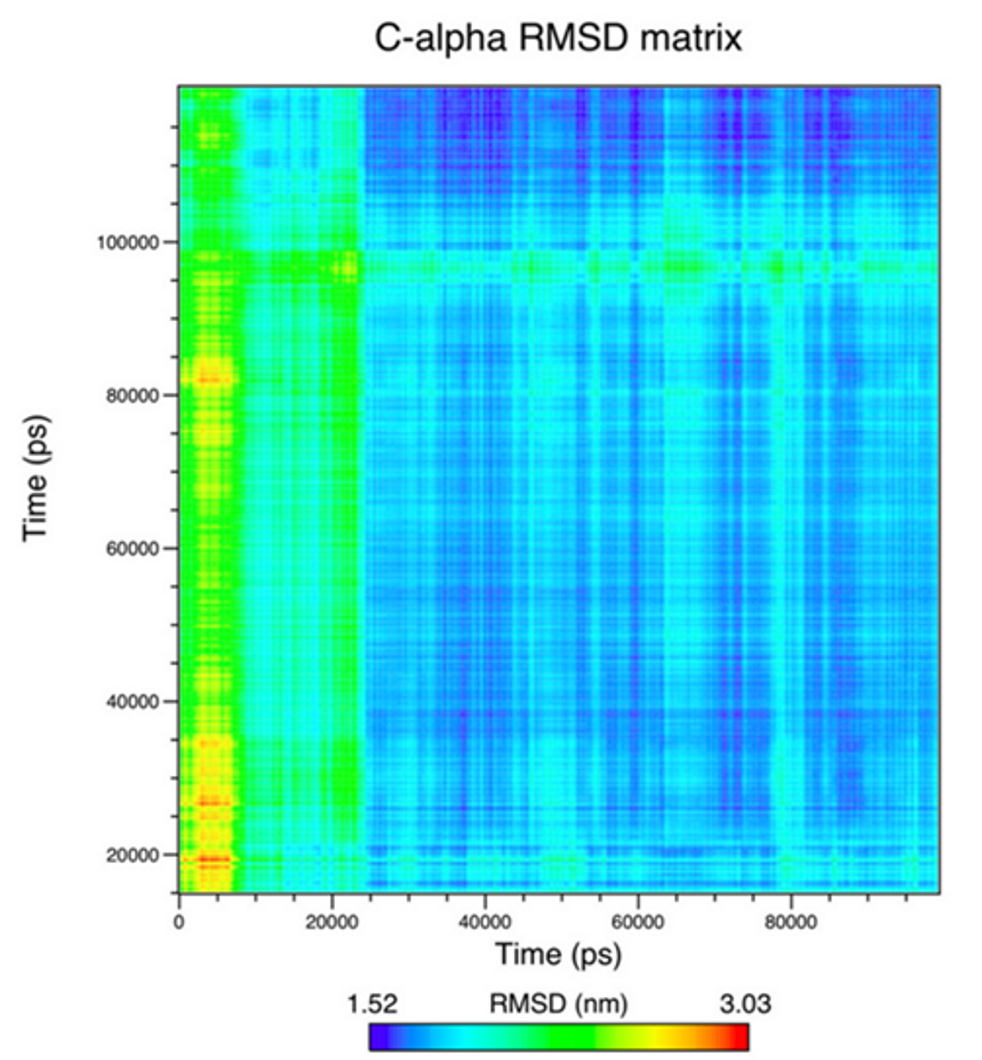

Supplement: Figure S6 — RMSD map. Map of time dependent RMSD comparison (snapshot to snapshot RMSD calculated on proteins Cα) between the III MD run starting from ATP-bound closed DnaK complex and the I MD run starting from ADP-bound open DnaK complex, showing an RMSD decrease towards the end of the trajectory, hence a convergent MD evolution to intermediate, semi-open similar structures. (TIFF) [file pcbi.1002844.s006.tiff]

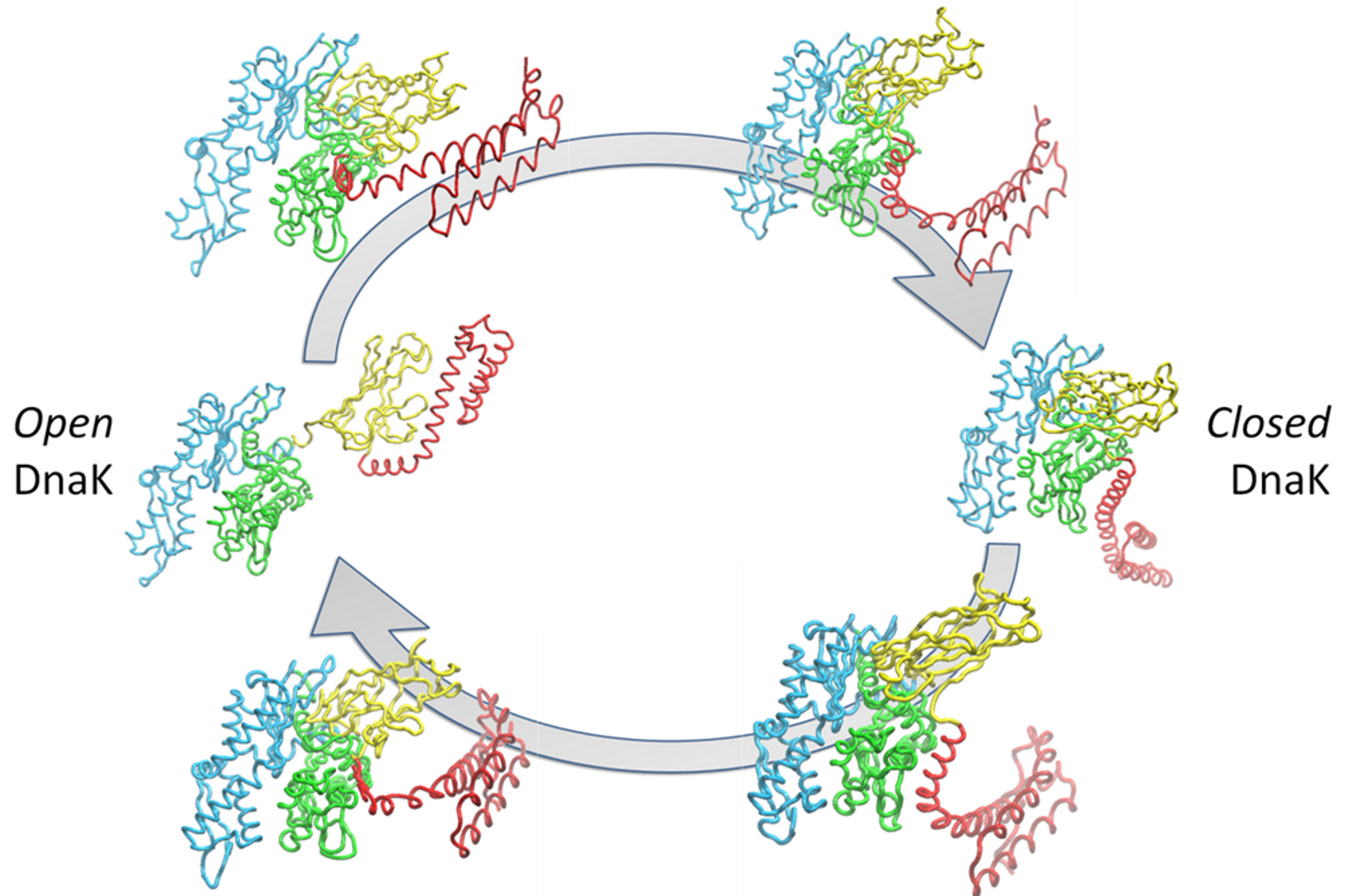

Supplement: Figure S7 — Transition pathway connecting the two DnaK open and closed states. Snapshots are extracted from the steepest-descent paths connecting open and closed DnaK structures, as obtained by applying the AD-ENM Web server (see main text). NBD lobe I is colored in green, lobe II is in light blue, βSBD is shown in yellow and αSBD is in red. (TIFF) [file pcbi.1002844.s007.tiff]

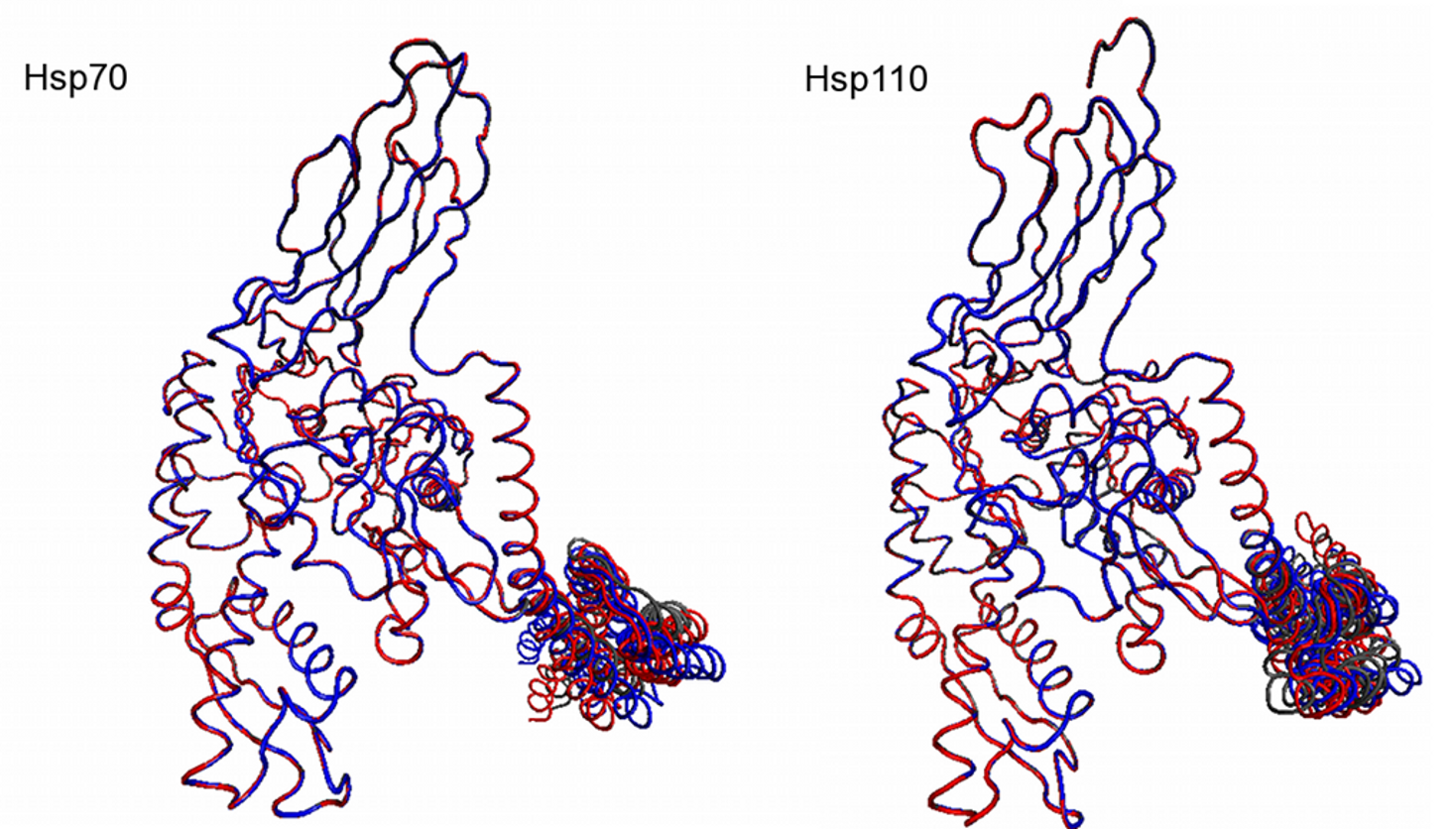

Supplement: Figure S8 — Normal mode analysis. ENM-based analysis of homology modeled open DnaK (here referred to as Hsp70) and of open Sse1 (Hsp110). By superimposing extreme structures as obtained by AD-ENM Web server the motion along the first three normal modes (first, blue; second, grey; third, red) is shown (see main text). The sequence stretch 500–527 in Hsp110 was removed from the analysis to improve comparison. (TIFF) [file pcbi.1002844.s008.tiff]
