# Supplementary material for: Molecular Mechanism of Allosteric Communication in Hsp70 Revealed by Molecular Dynamics Simulations
Source: PLoS Comput Biol. 2012 Dec 27;8(12):e1002844. doi: 10.1371/journal.pcbi.1002844 (PMC3531320; doi:10.1371/journal.pcbi.1002844)
Supplement: Table S1 — Trajectory clustering results. The number of clusters obtained fitting on Total, NBD or SBD residues is specified in brackets, the percentage represents the number of conformations belonging to the most populated cluster. (DOCX) [file pcbi.1002844.s009.docx]

**Table S1 Trajectory clustering results.** The number of clusters obtained fitting on Total, NBD or SBD residues is specified in brackets, the percentage represents the number of conformations belonging to the most populated cluster.

|  | | Total  (cutoff 0.5 nm) | NBD  (cutoff 0.5 nm) | SBD  (cutoff 0.5 nm) | Binding site (cutoff 0.2 nm) |
| --- | --- | --- | --- | --- | --- |
| *closed* DnaK | **ADP** | (7) 52% | (1) 100% | (7) 51% | (4) 52% |
|  | **ATP** | (18) 34% | (1) 100% | (10) 43% | (7) 62% |
| *open* DnaK | **ADP** | (22) 29% | (1) 100% | (1) 100% | (24) 24% |
|  | **ATP** | (15) 42% | (1) 100% | (4) 85% | (17) 41% |
| *open* Sse1 | **ADP** | (6) 70% | (1) 100% | (1) 100% | (7) 51% |
|  | **ATP** | (8) 61% | (1) 100% | (1) 100% | (17) 40% |
