# Supplementary material for: Molecular Mechanism of Allosteric Communication in Hsp70 Revealed by Molecular Dynamics Simulations
Source: PLoS Comput Biol. 2012 Dec 27;8(12):e1002844. doi: 10.1371/journal.pcbi.1002844 (PMC3531320; doi:10.1371/journal.pcbi.1002844)
Supplement: Table S2 — Persistence of the most populated hydrogen bonds during the single trajectories. Percentage of presence, during the single simulation, for each hydrogen bond involved in the identified network. (DOCX) [file pcbi.1002844.s010.docx]

**Table S2 Persistence of the most populated hydrogen bonds during the single trajectories.** Percentage of presence, during the single simulation, for each hydrogen bond involved in the identified network.

| H bond | Closed DnaK | | Open DnaK | |
| --- | --- | --- | --- | --- |
|  | ATP | ADP | ATP | ADP |
| Nucleotide-K67 | 74%  53%  50% | 43%  81%  4% | * | 96%  1%  18% |
| K67-E168 | 36%  25%  56% | 51%  49%  50% | 87%  74%  100% |  |
| E168-V139 | 100%  93%  94% | 92%  94%  97% | 87%  4%  65% |  |
| V139-I166 | 100%  95%  94% | 100%  94%  94% | 94%  81%  95% |  |
| I166-I137 | 100%  98%  91% | 100%  98%  96% | 67%  73%  83% |  |
| I137-R164 | 92%  70%  83% | 59%  90%  67% | 55%  79%  73% |  |
| Nucleotide-G194 | 30%  30%  33% | 76%  24%  30% | 100%  25%  100% | 97%  22%  40% |
| Nucleotide-G195 | 74%  98%  44% |  | 45%  4%  100% |  |
| Nucleotide-T196 | 94%  81%  100% |  | 49%  1%  82% |  |
| Nucleotide-G339 | 26%  18%  47% |  | 27%  30%  29% |  |
| G339-R342 | 51%  74%  63% |  | 66%  45%  39% |  |

*: ATP-R72 (70%, 52%, 68%); G71-K67 (29%, 32%, 29%)
